# Hall effect thruster impedance characterization in ground-based vacuum test facilities

**DOI:** 10.1007/s44205-024-00088-9

**Published:** 2024-12-02

**Authors:** David R. Jovel, Janice D. Cabrera, Mitchell L. R. Walker

**Affiliations:** School of Aerospace Engineering, Georgia Institute of Technology, Atlanta, GA 30332 USA

**Keywords:** Hall thrusters, Impedance, Electrical facility effects, Hall thruster AC characteristics

## Abstract

Hall effect thrusters (HETs) are typically regarded as DC electric propulsion devices as they are operated with isolated DC power supplies. However, it is well known that the HET’s discharge current possesses oscillations of varying magnitudes and frequencies and is thus a function of time with AC characteristics. The observed oscillations are caused by plasma processes associated with ion, electron, and neutral particle dynamics that occur inside the HET’s discharge channel and in the plume as the HET electrically interacts with its local operating environment. The extent to which plasma oscillations impact HET discharge dynamics is difficult to quantify due to the complexity of analyzing AC signals, given that the HET is a nonlinear, time-variant electrical load. In this work, we overcome the challenge of nonlinearity and time-variance of HETs by conducting a small-signal impedance analysis to characterize the effective resistance and reactance of the HET discharge with a novel and versatile impedance measurement diagnostic. The impedance magnitude and phase of a 7-kW class HET were measured from 100 Hz to 300 kHz with an excitation signal of ± 2 *V*_*pk*_ for two discharge operating conditions on krypton: 4.5 kW, 15 A and 6 kW, 20 A. The results were used to quantify resistive, capacitive, and inducive characteristics present within the HET discharge signature. For the 4.5 kW, 15 A thruster operating condition, the breathing mode capacitance was estimated to be 12.6 *µ*F with an inductance of 15.3 *µ*H. Furthermore, the impedance characteristics of the breathing mode are within ± 2.4 kHz of the power spectral density plots independently generated by time-resolved oscilloscope traces indicating good agreement in the frequency domain. Thus, the impedance measurement tool is a new diagnostic for characterizing the impedance and associated AC characteristics of HETs.

## Introduction

Since the 1970s, HETs have dominated the in-space electric propulsion (EP) industry as more than 500 thrusters have been operated in space supporting mainly station-keeping and electric orbit-raising maneuvers [1]. Currently, HETs comprise about 60% of all research activities in academia in the U.S. and abroad [2]. Moreover, HETs continue to be the primary propulsion option for many commercial and civil spaceflight programs [1, 2]. For such programs, the performance of HETs must be characterized inside ground-based vacuum test facilities through a rigorous flight qualification test campaign prior to operating them in space.

The primary performance parameters of HETs are thrust, specific impulse, lifetime, and efficiency at a fixed DC operating voltage and time-averaged discharge current for a given propellant. However, it is well known within the community that the discharge current is time-varying, indicating that the thruster possesses dynamic characteristics, and a function of user-defined operating parameters such as magnetic field strength and mass flow rate. For example, periodic discharge current oscillations such as the “breathing mode”, as observed by many thruster operators in the community, are dynamic characteristics of HETs. These discharge current oscillations are related to various complex physical processes, such as ionization events that occur inside the thruster’s discharge channel, during thruster operations. Moreover, these oscillations are observed to fluctuate about a mean value that serves as the time-averaged, DC discharge current value when discussing HET performance. Thus, we can regard the time-varying discharge current signal measured during thruster operation to be the superposition of the DC current value, *I*_*dis, dc*_, and the AC component, *I*_*dis, ac*_(*t*), consisting of the observed oscillations.

The extent to which the dynamic behavior of HETs is characterized in ground-based tests is based on time-resolved oscilloscope measurements that capture peak-to-peak discharge current, *I*_*dis, pk2pk*_, root-mean-square (RMS) discharge current, *I*_*dis, rms*_, and the frequency content embedded in the discharge current signal’s trace. The current waveform in the time-domain is converted into the frequency domain using a Fast Fourier Transform (FFT) algorithm. Once the signal is in the frequency domain, power spectral density (PSD) plots can be used to interpret the energy distribution of the signal across a frequency range of interest. The frequency content provides insight into the dynamic characteristics of HETs that are important for their stable operation in space. For example, sharply peaked energy content centered around a narrow frequency band may be used to inform thruster setpoints (i.e., magnetic field strengths or mass flow rate) for improved energy distribution across a wider frequency band pursuant to a quiescent operating point. Furthermore, the frequency content is used for the design and manufacturing of the power processing unit (PPU) that is used to power the HET in space. Thus, time-resolved oscilloscope measurements are essential and the community-accepted approach for characterizing the dynamic characteristics of HETs with no alternative options.

The motivation for this work is to supplement the existing method for collecting and interpreting time-resolved discharge current measurements by using impedance spectroscopy. To do so, we must first introduce the concept of impedance, the benefits such a measurement brings, and how impedance measurements can overcome the limitations of oscilloscope-based measurements. First, impedance is an electrical load’s total opposition to a time-varying current and voltage. Since the HET’s discharge current possesses AC characteristics, the HET discharge behaves as a time-varying electrical load with an impedance, *Z*_*dis*_. The impedance signature of an electrical load consists of a real resistance and imaginary reactance that captures the effects of time-varying electric- and magnetic-field phenomena contained within the load. These phenomena represent capacitive and/or inductive effects that can be useful in describing the HET’s dynamic behavior in the frequency domain. Quantifying these reactive effects can enhance our understanding of the physical processes inside the HET discharge and how they electrically interact with its operational environments. However, insight into these effects is limited as HETs are seldom portrayed as possessing time-varying electrical characteristics with properties that can be represented by a frequency-dependent impedance. Therefore, impedance spectroscopy can provide a different perspective in interpreting the dynamic behavior of HETs.

The phase, the angular relationship between the measured voltage and current signals in AC circuits, must be obtained to decompose impedance into its resistive and reactive elements [3]. In practice, this consists of injecting a reference voltage signal of known frequency across the electrical load, and measuring the current response, to provide a means for calculating the time offset between the two signals [4]. Collecting raw voltage and current measurements of the HET discharge in the time-domain using high-speed oscilloscopes is insufficient to measure the phase angle because the two signals are unsynchronized and not properly referenced to one another. Fortunately, equipment like impedance analyzers are self-contained devices with the ability extract the load’s impedance magnitude and phase as a function of frequency. However, the measurement technique assumes that the electrical load behaves as a linear, time-invariant (LTI) electrical load. For nonlinear, time-variant complex loads, such as the HET discharge at a DC operating condition, other methods such as small-signal impedance analysis can be used to measure the impedance magnitude and phase of the load. Although impedance spectroscopy has been fully implemented in other fields, it has not been adopted by the EP community as an alternative method for characterizing the HET as a time-varying electrical load.

The objective in this work is to implement impedance spectroscopy and prove its utility as an alternative method to characterize the dynamic behavior of a HET. Specifically, we aim to quantify resistive, capacitive, inductive, and resonant features contained in the impedance signature of a 7-kW HET between 100 Hz and 300 kHz frequency band. Interpreting the HET’s discharge characteristics in this manner provides the EP community with a new way of relating physical plasma processes to electrical engineering circuit elements. To achieve this, we developed a novel impedance measurement tool to measure the impedance profile of the discharge of a 7-kW HET at two thruster operating conditions on krypton: 4.5 kW, 15 A and 6 kW, 20 A. Furthermore, we speculate that as the EP community moves forward in realizing the potential of high-power HETs greater than 6 kW, the increase in discharge current levels tested for flight will require such an alternative method to characterize their dynamic behavior.

The organization of this paper is done in the following manner. In section “AC analysis and impedance spectroscopy background”, we introduce the concept of impedance spectroscopy, from an electrical engineering perspective, and describe our approach in applying this new method to a HET via small-signal impedance analysis. Section “Experimental setup” presents an overview of the experimental setup consisting of the thruster, vacuum test facility, diagnostics, and calibration technique for measuring the impedance of the HET discharge. In section “Results”, we provide the impedance profiles of the 7-kW HET operating at the 4.5 kW, 15 A and 6 kW, 20 A discharge operating conditions. In section “Discussion”, we present a comparison between the PSD of the discharge current and the impedance magnitude profile and show they agree using statistical correlation. In addition, the impedance magnitude and phase profiles are used to quantify resistive, capacitive, inductive, and resonant characteristics at the two HET discharge operating conditions. Finally, section “Conclusion” contains the conclusion with a summary of our findings supporting the use of impedance as an alternative method to characterize the dynamic behavior of HETs.

## AC analysis and impedance spectroscopy background

In ground-based HET testing, an isolated DC power supply is used to apply a constant discharge voltage, *V*_*dis*_, between the anode and cathode body electrodes. The anode mass flow rate, $\dot{m}$_*a*_, and electromagnetic coil currents predominantly determine the discharge current, *I*_*dis*_, at a fixed *V*_*dis*_. Moreover, the HET electrical load is dynamic because of the various spatiotemporal processes governing the generation of charge inside the discharge channel, conductivity of the plasma and its interaction with the local operating environment, and neutralization of charge in the plume [5–7].Consequently, the discharge current is characterized by a time-averaged DC component, *I*_*dis, dc*_, and a time-varying AC component, *I*_*dis, ac*_(*t*). The summation of *I*_*dis, dc*_ and *I*_*dis, ac*_(*t*) yields the time-varying discharge current, *I*_*dis*_(*t*), typically observed in ground-based test campaigns. As discussed in section “Introduction”, the HET load possesses a frequency-dependent impedance profile based on the frequency content embedded in *I*_*dis*_(*t*). The impedance profile of the HET load can provide insight in the following areas: (1) distribution of electrical energy over a range of frequencies, with each frequency corresponding to a physical process in the HET discharge, (2) inductive and/or capacitive behavior across specific frequency bands, (3) identify resonant frequencies between the plasma and its local operating environment, and (4) constructing equivalent circuits for complicated loads such as plasmas. Thus, we provide sufficient background on the analysis of time-varying electrical signals and the concept of impedance in the following subsections. Once these electrical engineering concepts are defined, we describe our approach in applying these to HETs.

### AC analysis and impedance of electrical loads

One of the objectives of AC analysis is to understand the steady-state current response of electric circuits driven by sinusoidal voltage sources. A sinusoidal voltage source produces a voltage signal that varies sinusoidally with time, *V*_*ac*_(*t*). The sinusoid is uniquely defined by its maximum amplitude voltage, *V*_*pk*_, angular frequency, ω, and phase angle, *Φ*. We can express the time-varying voltage signal using Eq. 1 below.

1 $${V}_{ac}\left(t\right)={V}_{pk}\text{cos}\left(\omega t+\phi\right)$$

Analyzing the steady-state current response, *I*_*ac*_(*t*), of the circuit in the time-domain can be cumbersome because it requires solving a system of differential equations based on Kirchhoff’s current and voltage laws for a given circuit application. However, if the steady-state solution for *I*_*ac*_(*t*) is sinusoidal, with a frequency identical to the sinusoidal voltage source frequency, and lags the voltage signal by a constant angle, we can use phasors to analyze the response of the circuit in the frequency domain. The phasor concept is based on Euler’s identity and is a complex number that carries the maximum amplitude and phase information of a sinusoid [8]. The phasor representation of the voltage source and the current response of the circuit is given by Eqs. 2 and 3, respectively. Note that we have set the phase angle to be with respect to the voltage source and therefore is $\phi$ = 0° for ${V}_{ac}$.

2 $${\mathbf{V}}_{\mathbf{a}\mathbf{c}}={V}_{pk}\angle0^\circ$$

3 $${\mathbf{I}}_{\mathbf{a}\mathbf{c}}={I}_{pk}\angle\phi^\circ$$

**Fig. 1:**
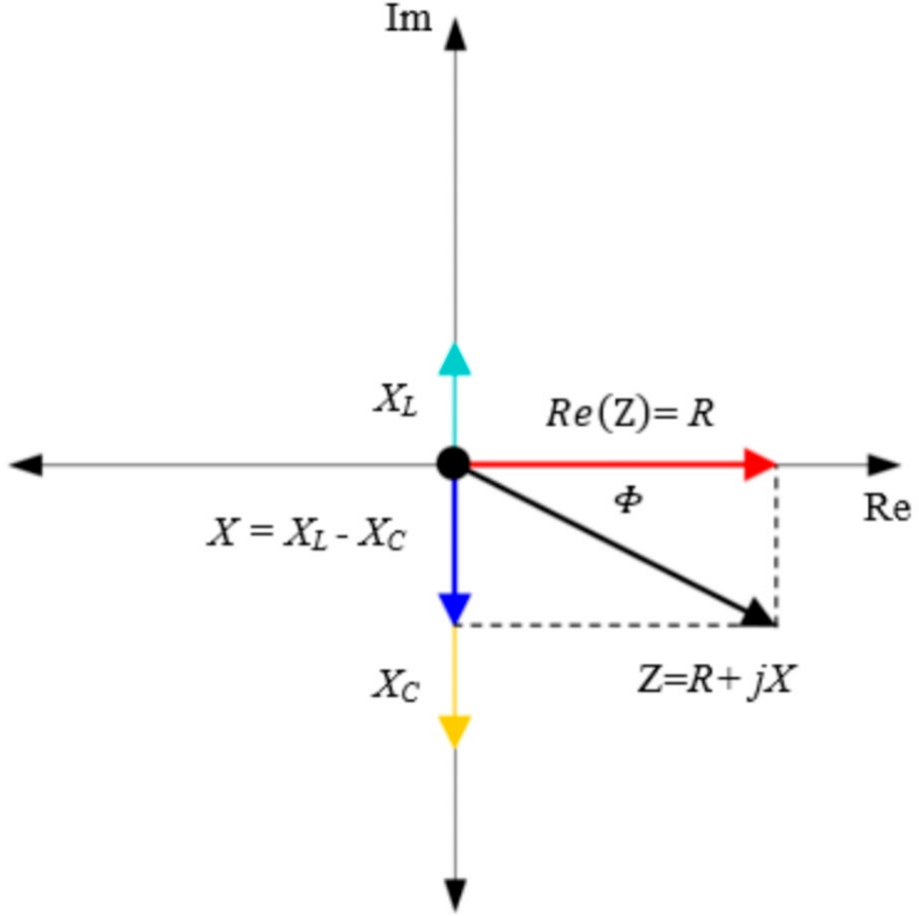
Impedance representation in the complex plane

Impedance is a complex quantity measured in Ohms, Ω, and is the combination of the real resistance, *R*, and imaginary reactance, *X*, observed at a particular frequency. In practice, the magnitude of impedance of an electrical load is readily calculated by computing the ratio of *V*_*pk*_ to *I*_*pk*_ for a given frequency [3, 9]. Whereas the phase of impedance is given by the phase angle difference between the voltage and current signals of the same frequency. Thus, the impedance of an electrical load has a magnitude and phase at a given frequency. The magnitude and phase of impedance are often represented in the complex plane as depicted in Fig. 1.

Analytical expressions for the complex impedance require that the electrical load be represented as an electrical circuit or an element of a circuit such as a resistor, *R*, capacitor, *C*, or inductor, *L*. For these cases, the utility of impedance in capturing the effects of time-varying electric- and magnetic field phenomena on the net flow of current through the load becomes readily apparent. For example, Ampere’s and Faraday’s Laws prove that a time-varying current will yield a net displacement of charge across capacitors and induce a voltage across inductors [8]. Thus, some of the current flowing through the load is consumed by these capacitive and/or inductive effects and establishes a net reactance that is only observed in time-varying AC applications. Due to these effects, the current and voltage waveforms are not synchronized in the time-domain and are offset from one another by a time constant. This time offset is equivalent to the phase angle, *Φ*, in the complex plane as previously discussed.

Reactance can be decomposed into inductive and capacitive reactances. Inductive reactance, *X*_*L*_(ω), and capacitive reactance, *X*_*C*_(ω), are both functions of frequency as determined by Eqs. 4 and 5, respectively. The sum of *X*_*L*_ and *X*_*C*_ yields the net reactance *X* as represented in Fig. 1. Many electrical loads can be described as a linear combination of circuit elements connected in series or in parallel depending on their application. For a generalized electrical load with resistive, inductive, and capacitive effects in series, the complex impedance, and its magnitude at angular frequency, *ω*, can be expressed by Eqs. 6 and Eq. 7, respectively. Whereas the phase is defined by the ratio of the imaginary net reactance, *X*, to the real resistance, *R*, components as given in Eq. 8.

4 $${X}_{L}\left(\omega\right)=j\omega L$$

5 $${X}_{C}\left(\omega\right)=\frac{-j}{\omega C}$$

6 $$Z\left(\omega\right)=R+j\left({X}_{L}-{X}_{C}\right)=R+j\left(\omega L-1/\omega C\right)$$

7 $$\left|Z\left(\omega\right)\right|=\sqrt{{R}^{2}+{X}^{2}}=\sqrt{{R}^{2}+{\left({X}_{L}-{X}_{C}\right)}^{2}}$$

8 $$\phi\left(\omega\right)={\text{tan}}^{-1}\left(\frac{X}{R}\right)={\text{tan}}^{-1}\left(\frac{{X}_{L}-{X}_{C}}{R}\right)$$

However, in many real electrical engineering applications, the equivalent circuit describing the load is rarely known and thus analytical expressions for impedance as a function of frequency are not readily available. Instead, techniques such as impedance spectroscopy are used to measure the impedance of the electrical load directly in the frequency domain. The general approach involves applying an electrical stimulus of known voltage and frequency to the load and observing the resulting current response [9]. The impedance is measured over a range of frequencies to give a complete profile of the load’s behavior in the frequency domain assuming it behaves as a linear, time-invariant (LTI) system. Once the magnitude and phase profiles of impedance are known, they can be used to decompose the measurements into resistive and reactive components to build a representative electrical circuit model composed of *R*’s, *L*’s, and *C*’s that adequately define the dynamic behavior of the electrical load. However, the stipulation of LTI is necessary in performing this successfully. The LTI condition is discussed in the following subsection and applied to HETs.

### Linear, time-invariant electrical loads

If an electrical load behaves as a linear and time-invariant (LTI) system, traditional methods such as Fourier transforms can be used to estimate the impedance of the load over a range of frequencies. Therefore, the system must satisfy the conditions of linearity and time-invariance. For a system to satisfy linearity, the transfer function between the input and output obeys the principles of homogeneity and superposition [10]. In this context, the input to an electrical load is the applied source voltage and the output is the resulting current through the load. Homogeneity, also called scaling law, requires that the transfer function scales proportionally between the input signal and output response. Thus, if the input signal was scaled by factor *A*, then the gain in the output response would be scaled by the same factor *A*. Ideal resistors, inductors, and capacitors are all examples of linear circuit elements [11]. The superposition property requires that the output response of the system to multiple input signals be the additive combination of the system’s response to the individual input signals. Thus, if the circuit is composed of individual linear circuit elements in series or parallel, then the circuit is linear, and its final state is the superposition of the individual responses of each element.

Next, we address the condition of time-invariance for an electrical load. Time-invariance, also called stationarity, requires that the transfer function between the input signal and the output response be constant in time [10]. In essence, the transfer function that describes the physical properties of the system does not change in time and therefore the response of the system to an input at time *t*_*1*_ is the same response if the same input is applied sometime later at time instant *t*_*2*_. Mathematically, this requires that the coefficients in the linear system of equations describing the electrical circuit are constant scalars that do not change in time.

Given the definition for LTI systems, we conclude that the HET plasma discharge is a nonlinear, time-variant electrical load. This is because the HET electrical load cannot be simply expressed as a combination of LTI elements. Perhaps a more obvious argument to prove the nonlinearity trait of a HET load is the fact that the load’s characteristics are completely determined by user-defined parameters such as $\dot{m}$_*a*_, $\dot{m}$_*c*_, magnet coil currents, propellant type, thruster geometry, and construction materials for a given *V*_*dis*_. Changing any of these parameters can result in a nonlinear change in the DC resistance characteristics of the thruster. Even in the simple case where only the mass flow rate is adjusted, the HET load can exhibit multiple DC resistance values for the same *V*_*dis*_. For example, the BHT-7000 tested in this work can achieve 30 Ω, 20 Ω, and 15 Ω equivalent DC resistances at a fixed 300 V discharge voltage bias. Furthermore, the HET discharge load is time-variant because of the characteristic time scales necessary to achieve steady operating conditions. For instance, it may require approximately 2 to 3 h for a 6-kW HET to achieve a steady state DC discharge current. Over the course of this time frame, the frequencies associated with important periodic phenomena, such as the breathing mode, are known to change [12]. Despite the fact that the HET is a nonlinear and time-variant load, electrical engineering methods such as small-signal impedance analysis exist to characterize the load within a small neighborhood centered around its DC operating point. This method is discussed in the next subsection.

### Small-signal impedance analysis for nonlinear, time-variant electrical loads

Small-signal impedance analysis is a common technique in electrical engineering that approximates the behavior of nonlinear electrical loads with an LTI model that is valid near the quiescent DC operating point of interest. A small AC excitation signal is superimposed onto the DC bias to perturb the load within the vicinity defined by *V*_*dc*_ ± Δ*V*_*ac, pk*_ and *I*_*dc*_ ± Δ*I*_*ac, pk*_. The magnitude of the excitation signal is chiefly governed by the application and usually experimentally determined. Generally, the signal should be small enough to perturb the response of the load such that its behavior is linearized about the DC operating point but larger than the observed noise floor so that it can invoke a measurable and repeatable response. For this work, a 2 V peak amplitude excitation signal, *V*_*pk*_, was sufficient to measure characteristics of the discharge impedance while minimizing the addition of energy into the system. The *V*_*pk*_ of 2 V constituted less than 1% of the 300 V DC bias observed during thruster operation while still invoking noticeable changes in the measured impedance. Many small-signal models have been developed for nonlinear electronic devices such as diodes, BJT transistors, and MOSFETs [13].

**Fig. 2:**
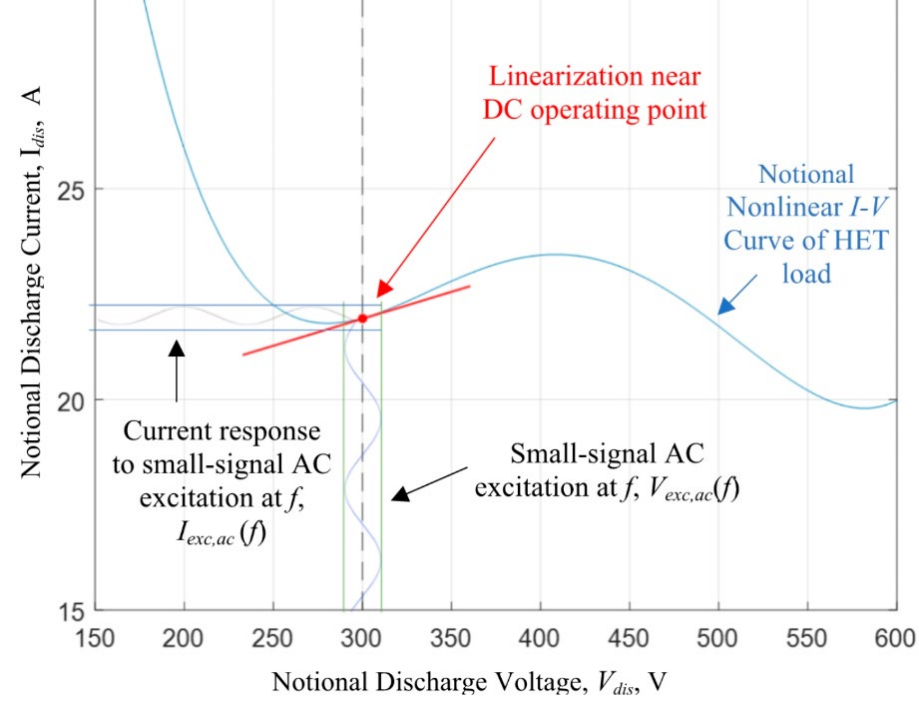
Small-signal impedance analysis for a nonlinear HET load with a notional discharge current vs. discharge voltage characteristic curve

Our approach to measuring the effective impedance of the HET discharge is based on small-signal analysis. The quiescent DC operating point is the discharge operating condition defined by the pair (*V*_*dis*_, *I*_*dis, dc*_). A discharge coupling circuit is used to superimpose an AC excitation signal of known frequency, *V*_*exc, ac*_(*f*), onto the DC bias. The corresponding current response of the HET discharge to the excitation signal, *I*_*exc, ac*_ (*f*), is independently measured. The impedance magnitude is given by the ratio of the excitation voltage to the measured current response whereas the phase is measured by the time-delay between the peak values of the two associated signals per cycle. Moreover, if the impedance magnitude and phase are measured over a frequency range of interest, then a complete profile of the dynamic behavior of the HET discharge is collected for this DC operating point. An illustration of the small-signal impedance analysis carried out in this work is provided in Fig. 2.

The resulting small-signal impedance profile is interpreted as a localized LTI description of the HET load at its DC operating point. The LTI description of the load is then decomposed into capacitive, inductive, and/or resonant effects and further associated with the physical processes observed in the thruster’s discharge channel and the coupling between the HET plume-and its local operational environment. In doing so, we implicitly assume that the HET load does behave as an LTI system within the excitation signal vicinity. Regarding the time-invariance property, we will use the time-averaged DC discharge current to confirm that the thruster has achieved thermal steady state prior to characterizing the impedance of the discharge. In this work, thermal steady-state is defined to be the condition where the change in time-averaged DC discharge current is less than 0.05 A/min.

## Experimental setup

In this xy, we provide an overview of the thruster, vacuum test facility, and diagnostics employed for this experiment. The objective of this section is to familiarize the reader with the experimental setup for this work.

### Test article

**Fig. 3:**
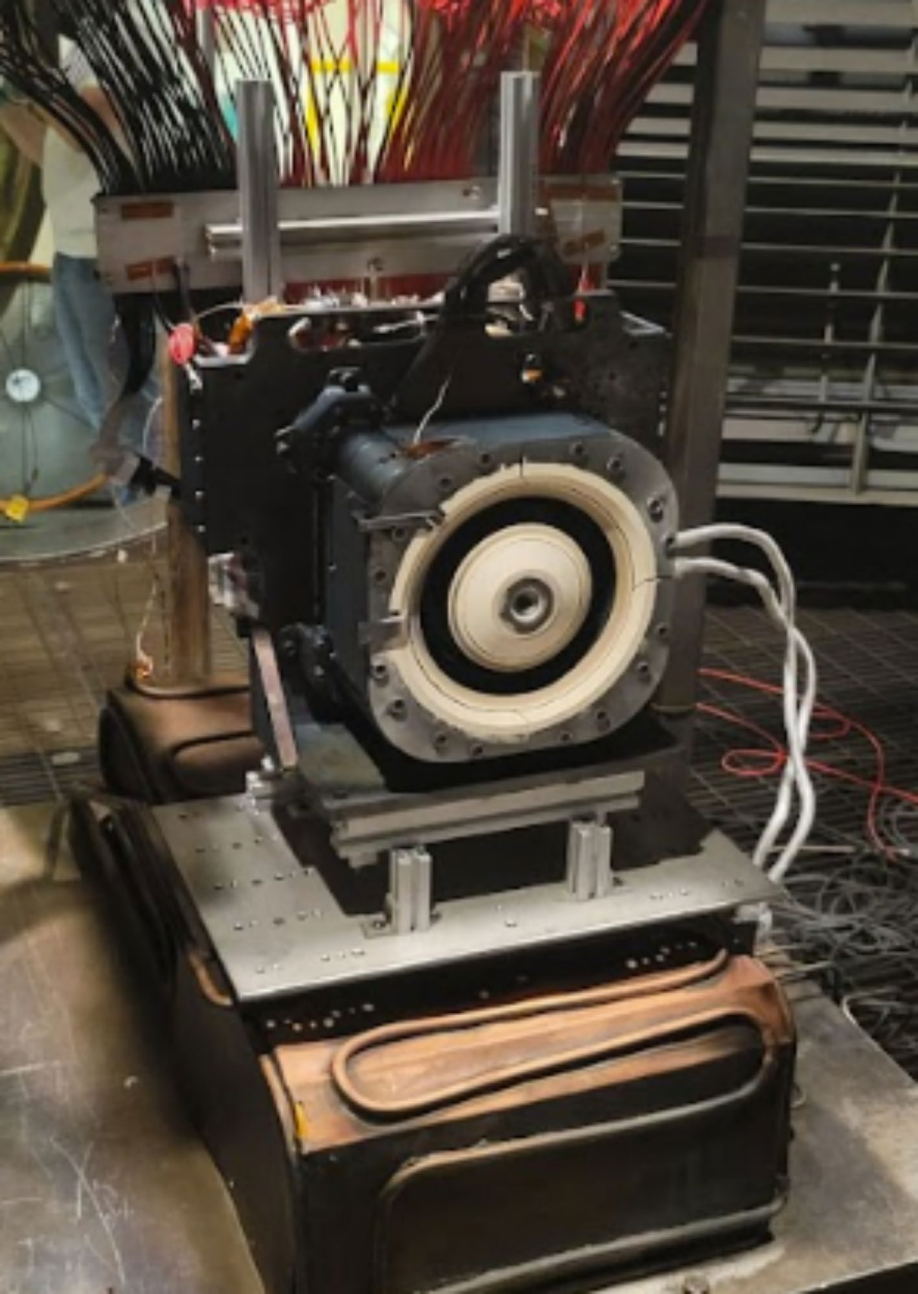
The BHT-7000 Hall effect thruster

The HET operated in this work is the Busek Hall Thruster 7000, BHT-7000. The BHT-7000 is based upon the evolution of the BHT-5000 thruster and is the latest design version in the moderate power class of HETs greater than 6 kW. Its predecessor, the BHT-6000, was selected by NASA to support Gateway’s Power & Propulsion Element and is currently undergoing flight qualification acceptance testing with a planned launch date in 2025 [14]. The BHT-7000 utilizes a center-mounted, 50-A barium-oxide cathode. An image of the BHT-7000 test article is provided in Fig. 3.

**Table 1:** BHT-7000 discharge operating conditions on krypton

Discharge power operating condition	V_dis_ (V)	I_dis, dc_ (A)	$\dot{m}$_anode_ (mg/s)	$\dot{m}$_cathode_ (mg/s)	I_mag_ (A)
4.5 kW	300 V	15 A	12.28	0.86	3
6 kW	300 V	20 A	15.39	1.08	3

For this work, the BHT-7000 was operated on krypton at the two discharge operating conditions shown in Table 1. The first thruster operating condition was 4.5 kW at 300 V, 15 A. The 4.5-kW discharge condition was selected because it is currently the state-of-the-art for high-power HETs with flight heritage [1]. The second discharge operating condition was 6 kW at 300 V, 20 A and was selected to measure the dynamic behavior of increased discharge current levels.

The thruster discharge was operated using a Magna-Power TS800-54 50 kW DC power supply. An RC filter using a high-power 0.533-Ω resistor bank and 100-*µ*F capacitor is installed between the DC supply and the HET power lines to protect the supply from the reflected discharge current oscillations. The anode and cathode flow rates were maintained at their respective values using commercially available MKS GE50A mass flow meters. Mass flow calibration was performed at these two flow conditions using a Mesa Labs DryCal 800 to ensure output flow uncertainty is less than ± 2%.

**Fig. 4:**
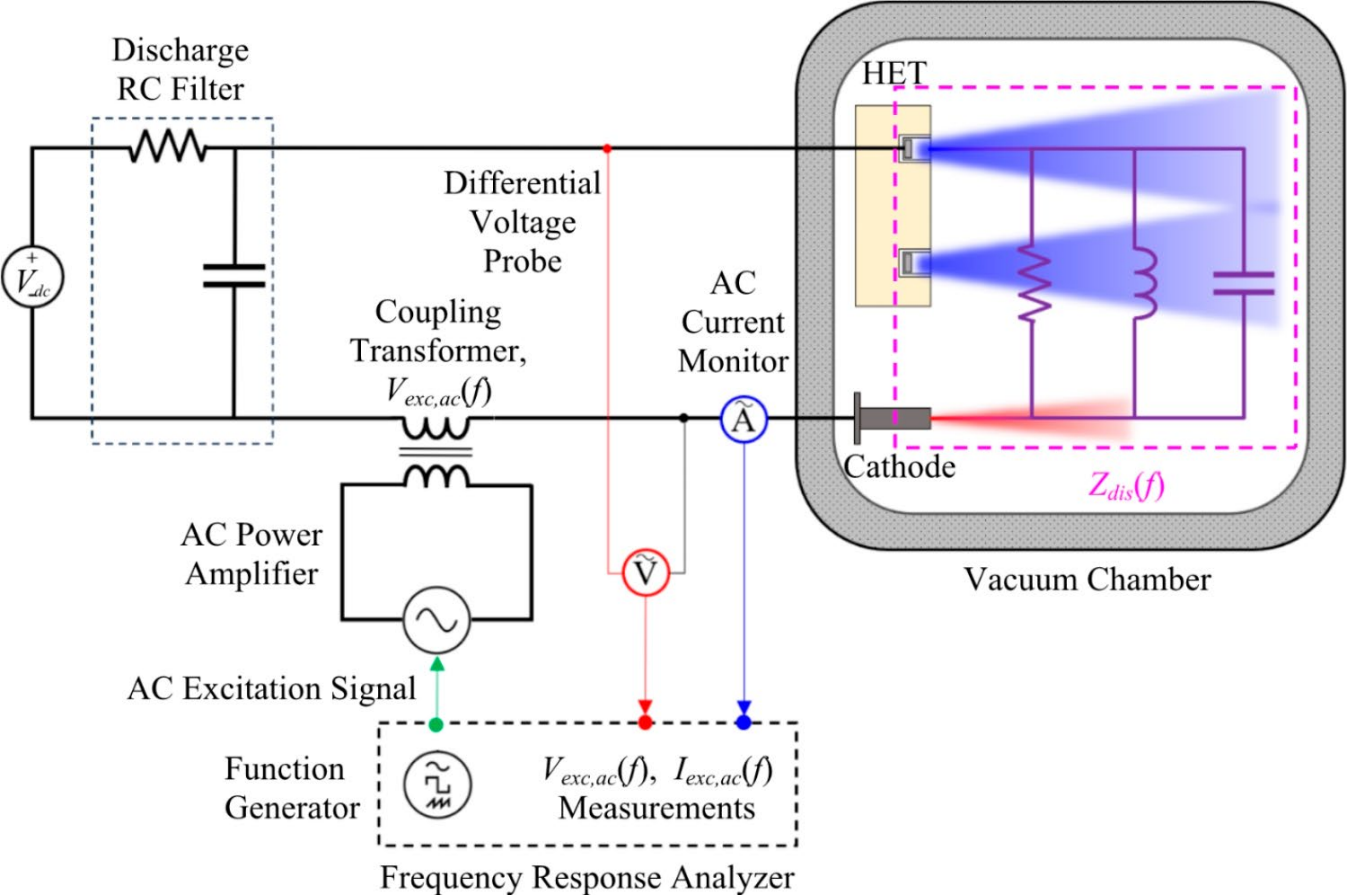
AC coupling circuit to perform small-signal impedance measurements on an energized DC discharge circuit

Time-resolved measurements of the discharge current and voltage were measured using a 1 GHz, 12-bit, up to 2.5 GS/s Teledyne LeCroy HDO6104 oscilloscope. The discharge current was measured using a Teledyne LeCroy CP150 current probe rated for DC up to 10 MHz whereas the discharge voltage was measured using a Powertek DP25 differential voltage probe rated up to 25 MHz and 1000 V *V*_*pk2pk*_. The discharge voltage probe was located at the vacuum chamber power feedthrough on the air-side and the discharge AC current monitor was placed before the discharge filter as shown in Fig. 4. Thruster-telemetry time traces observed on the oscilloscope were measured on the two channels simultaneously at a sampling frequency of 100 MS/s for a ± 50 ms collection interval.

### Vacuum test facility

The experiment was conducted at the Georgia Institute of Technology’s High-Power Electric Propulsion Laboratory in Vacuum Test Facility 2 (VTF-2). The facility is a stainless-steel cylinder with domed end caps measuring 4.6 m in diameter by 9.2 m in length. The chamber generates a high vacuum environment in two sequential stages. First, a Leybold RUVAC RA 5001 root blower backed by a single-stage, rotary vane SOGEVAC SV630 B mechanical pump brings the facility base pressure from atmospheric conditions to approximately 2.5 × 10^− 2^ Torr. Once a chamber outgas leak rate less than 0.1 mTorr/min is achieved, the blower and mechanical pump assembly is turned off. Then, 10 liquid nitrogen-cooled PHPK TM1200i cryopumps are activated, enabling the facility to achieve high-vacuum base pressures < 2 × 10^− 8^ Torr-N_2_ in approximately 24 h. Liquid nitrogen (LN2) is supplied to each cryopump in the temperature range between 90 and 110 K via vacuum-jacketed feed lines using a Stirling Cryogenics SPC-8 closed-loop, recirculating nitrogen liquefication system.

Three Varian 571 Bayard-Alpert hot filament ion gauges were used to measure the facility pressure at the chamber wall and 1-m away from the thruster exit plane. An Agilent XGS-600 controller was used to read out the ion gauge current measurements. The facility pressure readouts from the XGS-600 controller were collected digitally using LabView software at two data samples per second. The facility operational pressure, as measured by the internal gauge, was maintained below 5.6 × 10^− 6^ Torr-Kr for the 4.5 kW, 15 A test condition and below 8.2 × 10^− 6^ Torr-Kr for the 6 kW, 20 A test condition.

### Impedance measurement diagnostic

The primary diagnostic for this experiment is the frequency response analyzer used to measure the impedance magnitude and phase of the HET discharge at a fixed DC operating point. The main challenge was to superimpose the AC excitation signal safely and accurately onto the live discharge circuit conducting up to 20 A DC while adhering to the analyzer’s equipment specifications. To do so, an AC coupling circuit was designed to inject the AC excitation signal onto the DC discharge operating voltage. The AC coupling circuit used to perform the small-signal analysis for this work is shown in Fig. 4.

The three primary components of the AC coupling circuit were the frequency response analyzer (FRA), the power amplifier, and the isolation transformer. A Powertek Gain Phase 1700 Analyzer (GP1700) served as the FRA providing both the excitation signal to the load and real-time data processing of the measured AC voltage and current response of the HET discharge. The GP1700 is a self-contained test instrument with a frequency range of 10 *µ*Hz to 1 MHz and three isolated BNC channels: a function generator signal output channel and two sensing input channels. The unit has a frequency response analyzer setting that was used to sweep across the prescribed frequency range and measure the gain and phase at each frequency with 6-digit resolution. The GP1700 has a gain and phase measurement accuracy of ± 0.05 dB < 10 kHz and ± 0.02° < 10 kHz, respectively. The GP1700’s function generator signal was supplied directly to the AC power amplifier and Channel 1 measured *V*_*exc, ac*_(*f*) while Channel 2 measured *I*_*exc, ac*_(*f*). Additionally, the GP1700 has a built-function called ‘TRIM’ that allows the generator’s excitation output signal to be controlled within a 1% tolerance over the frequency sweep.

A model 7234 single-phase, four-quadrant power amplifier from AE Techron was used to amplify GP1700’s input signal and drive the AC current through the coupling circuit. The amplifier has a bandwidth of DC – 500 kHz at 15 V_rms_ and 14.8 A_rms_ mid-level power mode setting. The T2000 isolation transformer from AE Techron was used to couple onto the energized DC discharge circuit and inject the excitation signal. The transformer’s primary coil is connected directly to the 7234 amplifier whereas the secondary coil is connected in series with the low side of the DC power supply line corresponding to the cathode. The transformer has a frequency bandwidth of 10 Hz – 250 kHz within a 3dB roll-off. To compensate for the T2000’s roll-off characteristics, the GP1700’s TRIM function was used for the closed-loop control of the injected excitation voltage signal.

The AC voltage probe and current monitor used to quantify the impedance of the HET discharge are discussed next. The AC excitation voltage signal, *V*_*exc, ac*_(*t*), was measured using a Powertek DP25 high-voltage differential probe. A high-voltage probe was necessary because both the DC discharge bias and the AC signal are sensed for this measurement. A model 804 Pearson coil was used to measure the AC current response, *I*_*exc, ac*_(*f*). Both sensors were air-side and configured to be within 40 cm of the feedthrough on the power lines corresponding to the anode and cathode. Thus, the reference plane for the impedance measurement of the HET load was at the feedthrough and included the effects of the vacuum-side wire harness that extended from the feedthrough up to the thruster connections. A calibration procedure to account for the impedance of the vacuum-side harness is provided in the next subsection.

The frequency range for this test campaign was 100 Hz – 300 kHz and was based on the time scale of the physical processes inherent to HETs as well as performance limitations of the measurement components. The kilohertz range between 10 and 100 kHz was of interest because the dominant breathing mode frequency typically resides in this range as measured in past experiments. For the BHT-7000, the breathing mode was shown to be a function of the propellant used, discharge power, and magnet coil current and was expected to be between the ranges of 10–30 kHz. Higher frequencies up to 200 kHz are known to exist. Furthermore, applying an FFT on oscilloscope data of the discharge current shows that most of the energy content is contained within the DC – 500 kHz band. As a reference, the power spectral density of the H9 thruster reveals that the energy content drops three orders of magnitude in the range 3 kHz to 300 kHz [15]. In addition, the rated performance bands for many of the components used in the AC coupling circuit also placed a limitation on both the lower and upper frequency range. The T2000 has a rated performance up to 250 kHz limiting the upper range, whereas the Pearson coil showed degraded performance below 100 Hz, limiting the lower range.

We acknowledge the challenge of using this approach to discern between the current response associated with the excitation voltage signal and the time-varying discharge oscillations inherent to HETs. As noted previously, the HET discharge exhibits AC characteristics although powered by DC power supplies. These oscillations are present during the frequency response sweep that collects *V*_*exc, ac*_(*f*) and *I*_*exc, ac*_(*f*) over the prescribed range. The unique response to the excitation can be isolated from the inherent oscillations via digital phase-detection and digital filtering techniques. A digital lock-in amplifier and discrete Fourier transform (DFT) in the neighborhood of the injected frequency can be implemented to reject the naturally occurring harmonics of the discharge to identify the *I*_*exc, ac*_(*f*) if the reference signal is known. In this application, the reference signal was the AC excitation voltage signal, *V*_*exc, ac*_(*f*). To increase the performance of these built-in filters, the user can adjust certain settings to enhance accuracy of the measurement at each frequency. For example, the user can increase the number of reference signal cycles thereby increasing the data collection window which provides more data points to enhance the accuracy of the DFT results.

### Calibration procedure

A method to calibrate the vacuum-side wire harness and supporting electronics was developed for this work. The purpose of this calibration procedure was to quantify the impedance of the vacuum-side wire harness, chamber feedthrough, and electrical connections at the thrust stand that extend downstream from impedance measurement reference plane on the air-side. For this calibration procedure, a load of known impedance was required to replicate the two test conditions at 4.5 kW, 15 A and 6 kW, 20 A. To accomplish these two operating conditions, we assembled a high-power wire wound resistor bank consisting of three TE Connectivity 2,500 W, 33 Ω ± 5% and three Ohmite PFE5K1R60E 1100 W 1.6 Ω ± 10% resistors. The resistors were configured in a circuit to achieve the equivalent 20 Ω (300 V / 15 A) or 15 Ω (300 V / 20 A) DC resistances for the two operating conditions.

**Fig. 5:**
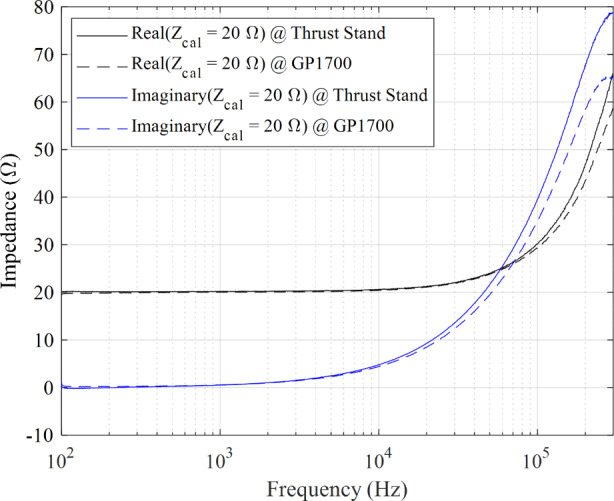
Calibration load impedance as a function of frequency for the 4.5 kW, 15 A test condition

The impedance magnitude and phase measurement profile of the calibration load were conducted across the 100 Hz – 300 kHz band at two locations and then compared. The first location was with calibration load installed directly at the thrust stand where the thruster resided during the test campaign. The second location was with the calibration load installed outside of the chamber at the defined reference plane near the GP1700 instrument. The impedance magnitude and phase between the two locations were compared to quantify the additional impedance of the wire harness and other supporting connections. Since the wire harness and connectors behave as LTI elements, their impedance contributions can be directly subtracted from the collected measurement scans to correct the profile of the HET discharge. This correction consisted of decomposing the measured HET impedance into its imaginary and real components and then subtracting the corresponding real and imaginary components obtained from the calibration procedure at the respective power level. The calibration datasets consistently revealed that the intermediate cabling between the feedthrough and the thrust stand added inductive effects of more than 15% for *f* ’s > 100 kHz. At the maximum range of 300 kHz, the wire harness provided an additional 14 Ω. A sample calibration dataset showing the difference in the real and imaginary components between the thrust stand and the GP1700 reference plane for the 4.5 kW, 20 Ω case is provided in Fig. 5. We note that the impedance for the resistor bank is not purely resistive as they are wire wound resistors with known inductance at *f* ’s greater than 10 kHz.

In addition, a frequency response sweep was performed on the Pearson coil current monitor and the DP25 differential probe to identify roll-off across the measurement band. Roll-off refers to the attenuation of a signal in terms of gain and phase across a frequency range. For the differential voltage probe, the GP1700 function generator applied a known excitation voltage at the sensing leads and was measured on Channel 1 whereas the scaled down output voltage signal was measured on Channel 2. The gain between output and the input was measured from 100 Hz − 300 kHz and exhibited a response of 1:200 ± 1% in that range. An 11 Ω ± 0.5% calibration resistor was used to calibrate the transfer function roll-off in gain and phase of the AC current monitor. For the model 804 sensor used in this work, a gain loss and phase offset of more than 3% and 13° was observed for frequencies less than 300 Hz. For frequencies above 2 kHz, the current monitor exhibited a gain loss and phase offset of less than 1% and 1.5°, respectively. We also characterized the roll-off of the T2000 coupling transformer and identified a gain loss of up to -2.3 dB for *f*’s > 200 kHz. However, we utilized the GP1700’s TRIM function for closed-loop control of the excitation voltage signal to compensate for the roll-off characteristics of the coupling transformer.

### Uncertainty analysis of impedance measurement technique

**Fig. 6:**
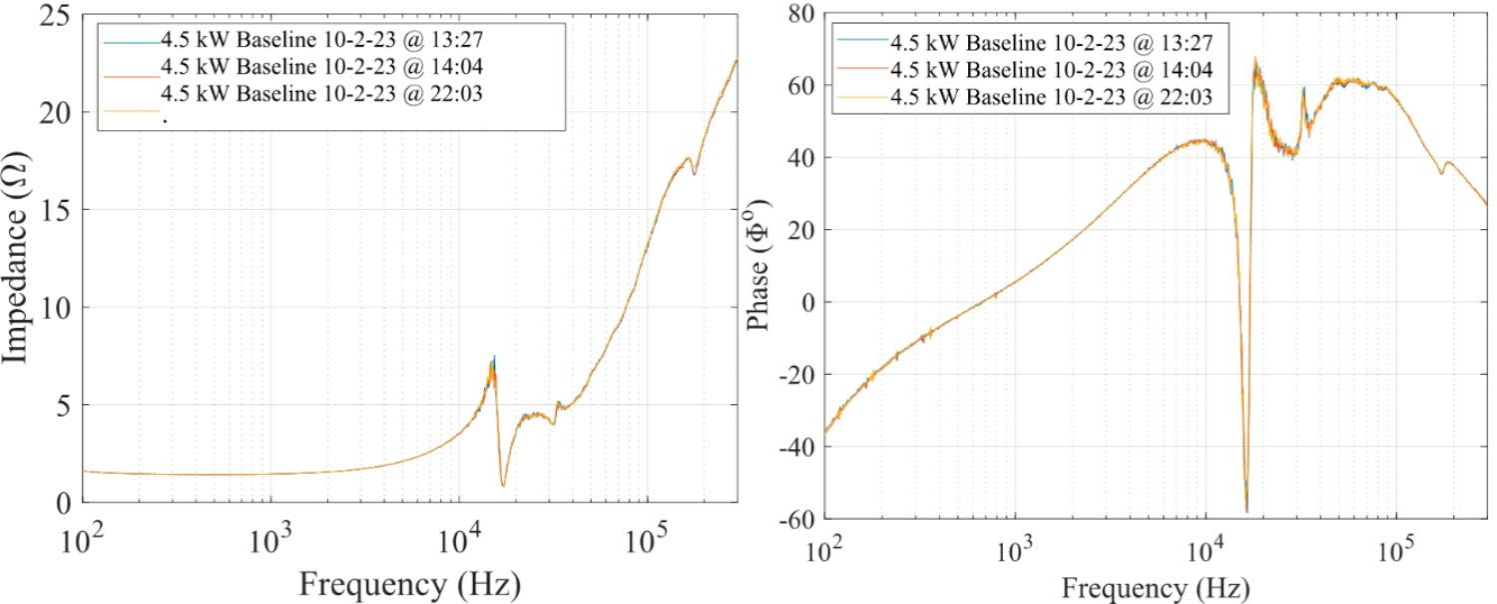
Impedance magnitude and phase at the 4.5 kW, 15 A test condition at various times

The uncertainty in the impedance data is attributed to random and systematic errors introduced by the equipment used to collect measurements and how the technique was implemented throughout the experiment. The sources of random errors are: (1) gain/phase measurement error inherent to the GP1700 FRA, (2) current measurement error inherent to the Model 804 AC current monitor, and (3) voltage measurement error inherent to the DP25 differential voltage probe. The uncertainty associated with the three error sources are due to stochastic fluctuations and can be reduced by taking successive measurements. For this reason, a minimum of three impedance scans from 100 Hz to 300 kHz were collected sequentially at a specific test condition. The mean value between three successive impedance sweeps was reported in this work. The standard deviation for each impedance data point was calculated to be less than 1% of the average impedance value using this approach. As an example, we show in Fig. 6 three impedance magnitude and phase measurement scans for the 4.5 kW, 15 A test condition after achieving thermal steady state to illustrate the repeatability of this measurement technique. We use the 24-hour clock system to denote the real time at which each scan was collected in the format HHMM. The blue curve was collected at 13:27, the orange curve was collected at 14:04, and the yellow curve was collected at 22:03. The eight-hour window between the orange and yellow curves was chosen to provide evidence of consistency and repeatability in capturing the impedance and phase characteristics of the HET discharge at the thermal steady state condition.

Systematic errors result from persistent issues inherent in the configuration and implementation of the measurement technique that bias the true value of impedance. The sources of systematic errors in this work are: (1) physical location of voltage and current probes for impedance measurements, (2) excitation voltage amplitude, and (3) steady-state operating condition of the HET. The first systematic error biases the true impedance of the HET plasma discharge by including the impedance of the wire harness extending from the measurement location to the anode and cathode connections at the thruster. The physical location of the voltage and current measurement probes was outside of the facility at the power feedthrough corresponding to the anode and cathode electrodes and thus introduced a systematic error in all impedance measurement sweeps. We addressed this systematic error by using the calibration load discussed previously to correct for the persistent bias in all measurements.

The second systematic error is based on the effect of the excitation voltage amplitude, *V*_*pk*_, on the measured current response of the HET discharge. We addressed this uncertainty experimentally by measuring the impedance after exciting the load with peak amplitudes: 0.5, 1, 2, 3, and 4 V. For the moderate discharge power levels tested in this work, the measured impedance was relatively impartial the choice of *V*_*pk*_ except for measurements near resonant frequencies centered around the breathing mode. We expect this sensitivity to be emphasized for lower-power HETs.

**Fig. 7:**
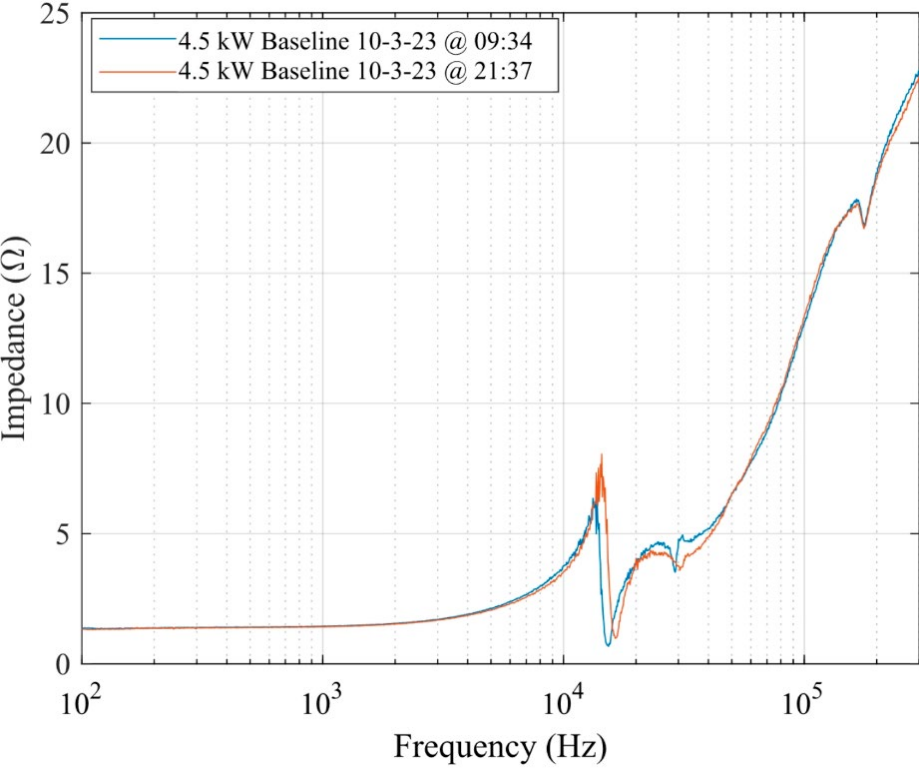
Impedance magnitude as a function of frequency for the 4.5 kW, 15 A test condition approximately 12 h apart

The systematic error associated with impedance measurements collected in the transient, warm-up phase before the thruster achieved steady-state operating conditions was the most impactful. In Fig. 7, we show two impedance scans obtained 12 h apart for the same 4.5 kW, 15 A test condition. The blue curve was collected at 09:34 and the red curve was collected at 21:37. The reader can see a clear distinction between the two traces, especially between 10 kHz and 50 kHz. Therefore, the time at which the impedance sweep was performed is an important factor when quantifying this systematic error. We corrected for this measurement bias by operating the HET for approximately 2 to 3 h until the time-averaged DC discharge current approached a steady value as defined in section “AC analysis and impedance spectroscopy background”. The impedance measurements obtained after the thruster achieved steady-state operating conditions are reported in this article.

## Results

In this section, we provide the results obtained from the work conducted for this manuscript. First, we present the average impedance magnitude and phase profiles for the 4.5 kW, 15 A from 100 Hz to 300 kHz. Then, we present the average impedance magnitude and phase profiles for the 6 kW, 20 A from 100 Hz to 300 kHz.

### Impedance magnitude and phase of the 4.5 kW, 15 A operating condition

**Fig. 8:**
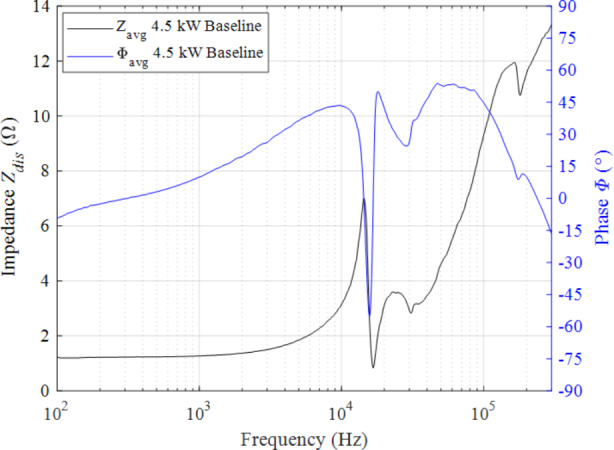
Impedance magnitude and phase as a function of frequency for the 4.5 kW, 15 A discharge operating condition on krypton

The impedance magnitude and phase profile for the 4.5 kW, 15 A discharge operating condition from 100 Hz to 300 kHz at a fixed excitation voltage of 2 V *V*_*pk*_ is shown Fig. 8. The magnitude of impedance is shown in black, and the phase is shown in blue. The impedance magnitude profile trends upward with various peak-and-trough pairs as frequency increases. The most notable peak is located at 14.32 kHz with an impedance of 7 Ω and was accompanied by a decrease in phase from 43.5° to -55.1°. Three successive rising peaks and troughs occur between 16 kHz and 300 kHz. The first is a low bulge with a center frequency of 24.62 kHz followed by a small, soft step with a frequency of 33.1 kHz. Impedance monotonically increases up to the third peak at 164.9 kHz with a value of 11.9 Ω. The phase transitions associated with the three successive peaks are not as pronounced as that of the first dominant peak.

### Impedance magnitude and phase of the 6 kW, 20 A operating condition

**Fig. 9:**
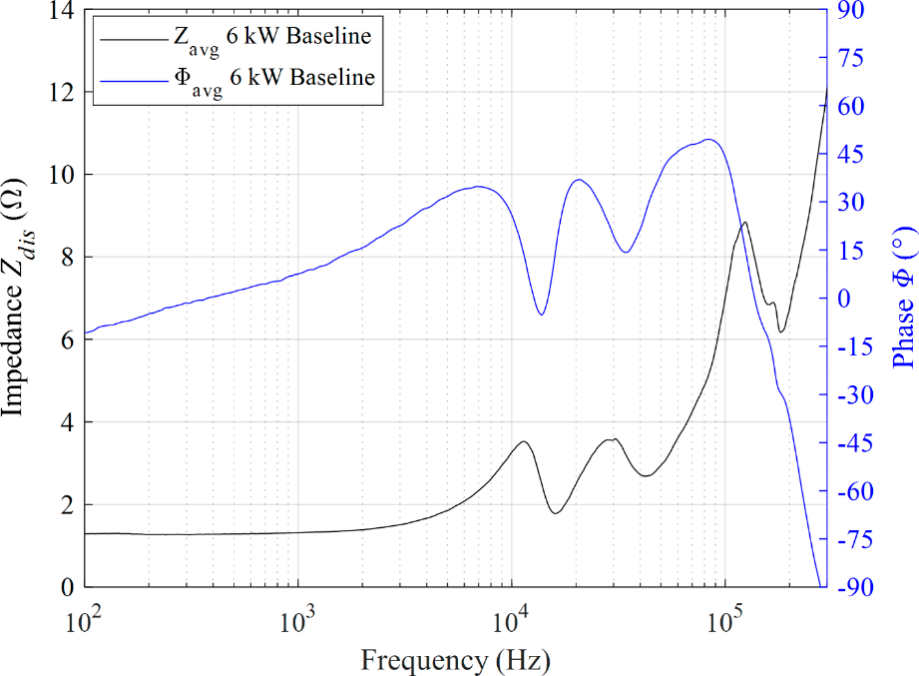
Impedance magnitude and phase as a function of frequency for the 6 kW, 20 A discharge operating condition on krypton

The impedance magnitude and phase profile for the 6 kW, 20 A discharge operating condition from 100 Hz to 300 kHz at a fixed excitation voltage of 2 V *V*_*pk*_ is shown in Fig. 9. The magnitude of impedance is shown in black, and the phase is shown in blue. The impedance magnitude profile trends upward with three main peak-and-troughs pairs as frequency increases. First, we address the two smooth humps observed between 6 kHz and 40 kHz. The first hump has a peak located at 11.43 kHz with an impedance of 3.53 Ω and was accompanied by a decrease in phase from 34.8° to -5.2°. The second hump has a peak located at approximately 29.5 kHz with an impedance value 3.56 Ω. The impedance magnitude monotonically increases culminating to a third, more distinguishable peak at 124.2 kHz with a value of 8.84 Ω. The phase transitions associated with the first two successive peaks are not as pronounced as that of the last peak which decreased precipitously from 49.5° to -97.6°.

## Discussion

In this section, we analyze the impedance profiles for the 4.5-kW and 6-kW discharge operating conditions. To begin, we compare the magnitude of impedance profile with the PSD of *I*_*dis*_(*t*) profile at each discharge operating condition. Then, we analyze the impedance magnitude trace of each power level separately and identify key frequency bands and comment on their resistive, capacitive, inductive, and resonant features. Furthermore, we compare and discuss the resistance and reactance profiles between 4.5-kW and 6-kW test conditions to conclude that the small-signal impedance characteristics are unique to thruster discharge operating conditions.

### Comparison between PSD and impedance magnitude profiles

**Fig. 10:**
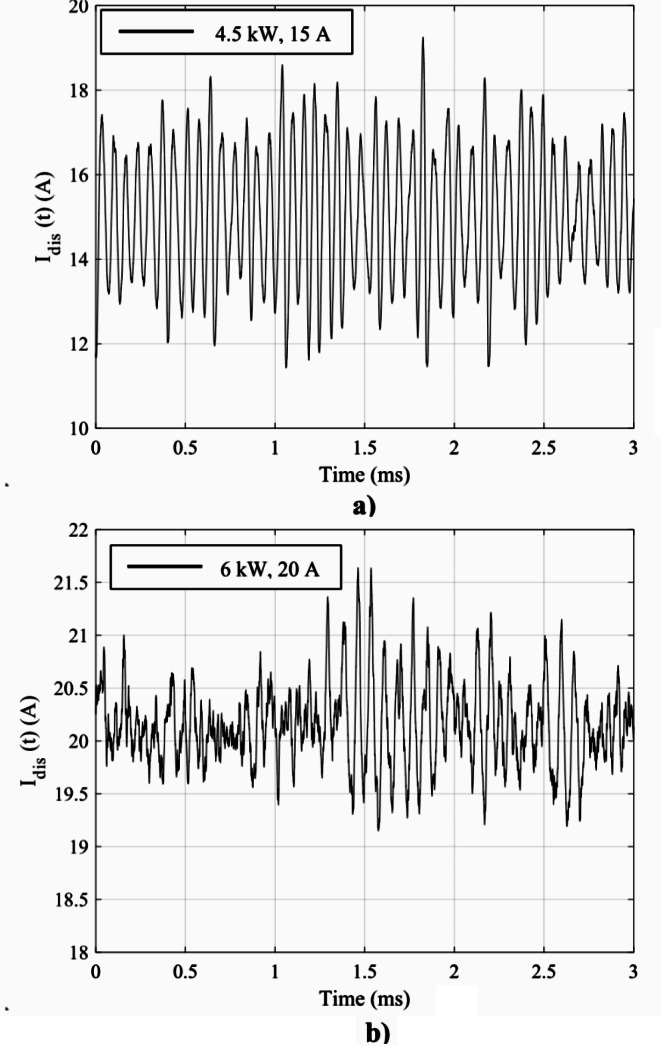
*I*_*dis*_(*t*) for the (**a**) 4.5 kW, 15 A test condition (**b**) 6 kW, 20 A test condition

**Fig. 11:**
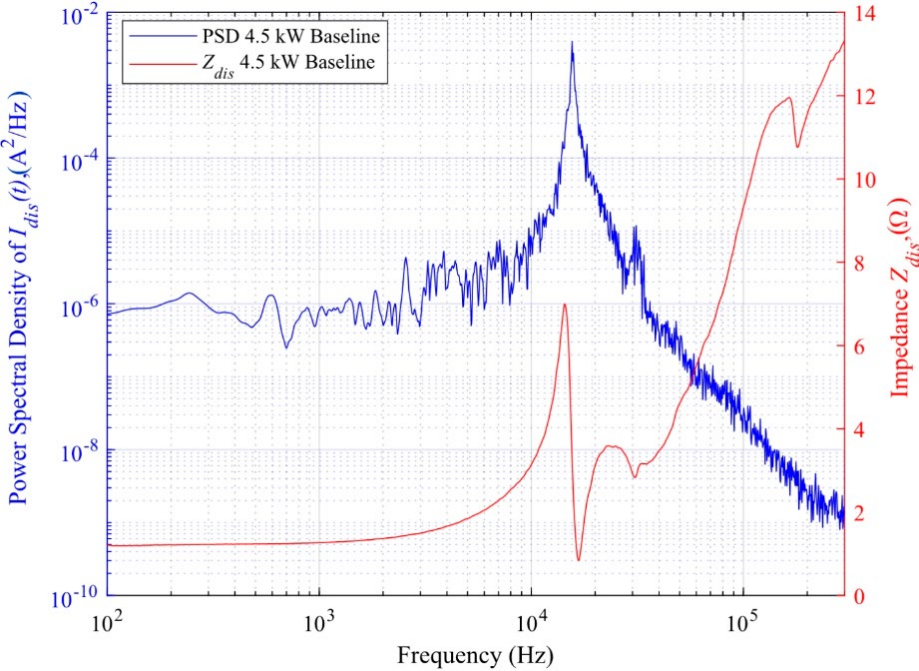
PSD of *I*_*dis*_(*t*) and impedance magnitude as a function of frequency for the 4.5 kW, 15 A test condition

**Fig. 12:**
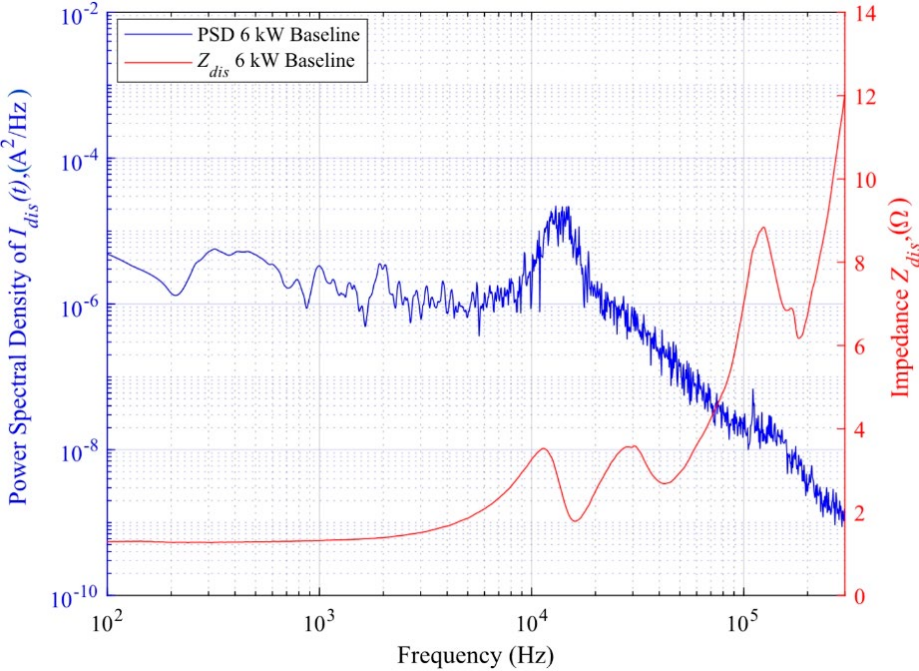
PSD of *I*_*dis*_(*t*) and impedance magnitude as a function of frequency for the 6 kW, 20 A test condition

In order to demonstrate the utility and promise of impedance as an alternative method to characterize the dynamic characteristics of HETs, we organize this subsection as follows. First, we present *I*_*dis*_(*t*) traces from the oscilloscope introduced in section “Experimental setup”. Figure 10a) displays *I*_*dis*_(*t*) at the 4.5 kW, 15 A test condition while Fig. 10b) displays *I*_*dis*_(*t*) at the 6 kW, 20 A test condition. Next, we convert each discharge current waveform to the frequency domain and obtain the PSD representation. Figure 11 shows the PSD of *I*_*dis*_(t) and impedance magnitude as a function of frequency for the 4.5 kW, 15 A test condition. Figure 12 shows the PSD of *I*_*dis*_(t) and impedance magnitude as a function of frequency for the 6 kW, 20 A test condition. In both figures, the red curve is the magnitude of impedance whereas the blue curve is the PSD of *I*_*dis*_(*t*). First, we identify the breathing mode frequency from the PSD plot at each condition. Next, we provide a brief visual comparison between the two profiles. Then, we provide the results of the statical correlation analysis performed to estimate the degree of association between the impedance magnitude and PSD of *I*_*dis*_(*t*) profiles.

The frequency with the largest peak in the PSD plot was assumed to be the frequency associated with the breathing mode, *f*_*BM*_. The assumption is reasonable as the energy content contained within ± 2 kHz of the observable peak comprises more than 30% of the total energy of the remaining signal. The energy distribution about the local peak is assumed to be normally distributed and a Gaussian fit is applied to identify the full-width, half-maximum (FWHM) range associated with the local peak frequency. In Fig. 11, the breathing mode frequency at the 4.5 kW, 15 A thruster operating condition from the PSD plot is 15.56 kHz ± 980 Hz. A second peak is observed at 31.24 kHz ± 900 Hz. In Fig. 12, the breathing mode frequency at the 6 kW, 20 A thruster operating condition is 13.74 kHz ± 1.1 kHz.

The PSD of *I*_*dis*_(*t*) and the magnitude of *Z*_*dis*_(*f*) plots at the 4.5 kW, 15 A test condition exhibit similar trends across different frequency bands. In Fig. 11, the two profiles gradually increase within the frequency band of 100 Hz to 14 kHz. They both rapidly increase to a peak near 15 kHz. The peak value for the *Z*_*dis*_(*f*) magnitude was 7 Ω at 14.32 kHz and for the PSD of *I*_*dis*_(*t*) the peak value was 4.1 × 10^− 3^ A^2^/Hz at 15.56 kHz ± 980 Hz. The statistical correlation in this band is 0.92 which indicates these two profiles are highly correlated within this frequency band. Given this strong correlation, we infer that the peak at 14.32 kHz in the *Z*_*dis*_(*f*) magnitude profile is associated with the breathing mode. In the frequency band of 15 kHz to 35 kHz, the impedance magnitude plot demonstrated a distinct reduction in impedance of 840% and rose to a pronounced hump at 24.62 kHz followed by a softer hump at 33.1 kHz. In contrast, the PSD profile exhibited a logarithmic decay with another peak located at 31.24 kHz ± 900 Hz. From 35 kHz to 300 kHz, the PSD of *I*_*dis*_(*t*) and the *Z*_*dis*_(*f*) magnitude plot trend oppositely with the PSD steadily decaying while the *Z*_*dis*_(*f*) magnitude profile rises monotonically. There is a final distinguishable peak in the *Z*_*dis*_(*f*) magnitude profile, with a value of 11.9 Ω located at 164.9 kHz, that is not present in the PSD curve. In the 35 –300 kHz band, PSD of *I*_*dis*_(*t*) and *Z*_*dis*_(*f*) magnitude are negatively correlated with a correlation coefficient of -0.96. We conclude the analysis for the 4.5-kW test condition with the identification of a highly correlated breathing mode frequency with the average value of 14.94 kHz.

Next, we compare the PSD of *I*_*dis*_(*t*) and the magnitude of *Z*_*dis*_(*f*) plots trends across different frequency bands for the 6 kW, 20 A test condition. In Fig. 12, the two profiles reach an initial peak within 12.5 kHz ± 1.25 kHz. The peak value for the *Z*_*dis*_(*f*) magnitude was 3.53 Ω at 11.43 kHz and for the PSD of *I*_*dis*_(*t*) the peak value was 2.2 × 10^− 5^ A^2^/Hz at 13.74 kHz ± 1.1 kHz. The two curves have a positive correlation of 0.5 in the frequency band of 1 –20 kHz. Compared to the 4.5 kW, 15 A thruster operating condition, the correlation in the frequency band corresponding to the first peak appears to be weaker. This may be due to the overall decrease in energy content stored within the discharge current oscillations inherent to 6 kW, 20 A due to its more quiescent-like behavior. This is supported by the difference in *I*_*dis, pk2pk*_ across the two conditions as the 4.5 kW, 15 A test condition has a *I*_*dis, pk2pk*_ of 5.45 A whereas the 6 kW, 20 A test condition has a *I*_*dis, pk2pk*_ of 1.26 A. Then, the *Z*_*dis*_(*f*) magnitude profile smoothly declines to a local trough with a magnitude of 1.8 Ω located at 16 kHz before gradually ascending to the second hump with a maximum value of 3.56 Ω located at 29.5 kHz. In contrast, the PSD profile reveals a logarithmic decay through 300 kHz. From 42 kHz onward, the magnitude of *Z*_*dis*_(*f*) trends opposite to that of the PSD plot. *Z*_*dis*_(*f*) achieved a third hump with a steep decrease with a local minimum of 6.18 Ω centered at 180.5 kHz. The two curves are negatively correlated with a coefficient of -0.85 within the band of 42 kHz and 300 kHz. We conclude the analysis for the 6-kW test condition with the identification of a fairly correlated breathing mode frequency with the average value of 12.59 kHz.

### Resistance and reactance of the 4.5 kW, 15 A operating condition

**Fig. 13:**
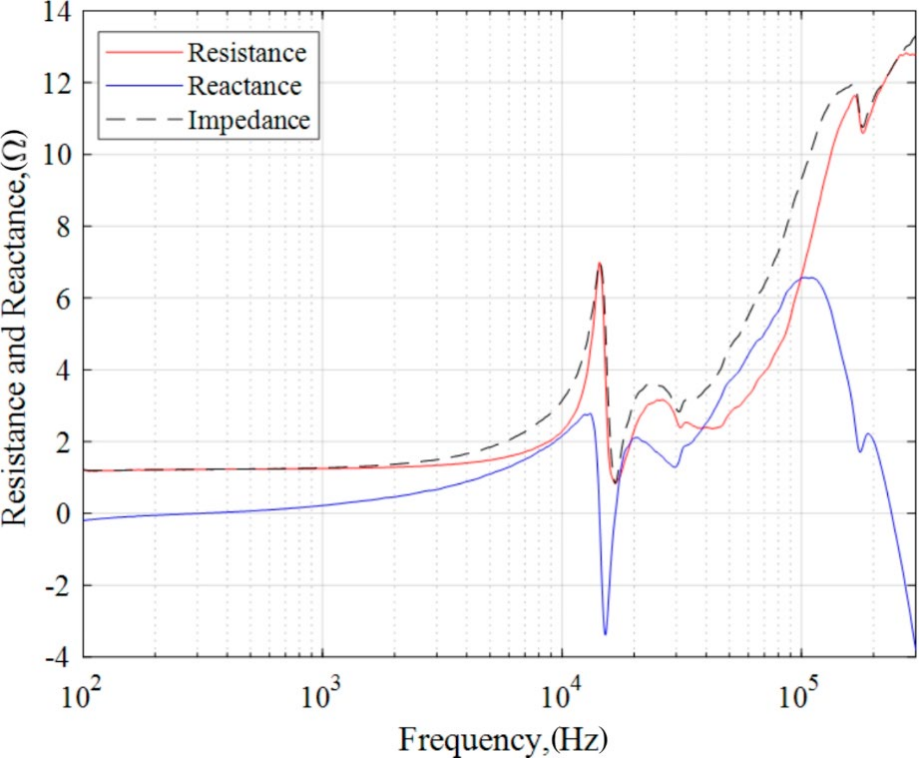
Resistance and reactance at the 4.5 kW, 15 A test condition

We decompose the measured impedance magnitude into its real and imaginary components using the phase information as provided by Eqs. 7 and 8. The real component is associated with resistance whereas the imaginary component is associated with reactance components as described in section “AC analysis and impedance spectroscopy background”. In Fig. 13, we plot resistance in red and reactance in blue for the 4.5 kW, 15 A discharge operating condition. For reference, we also include the net impedance magnitude in the figure as a dashed curve.

In the band 100 Hz – 1 kHz, the impedance was mostly characterized by a steady resistive value of 1.24 Ω with a small reactive component less than 0.22 Ω. In addition, the reactance was observed to be transitioning from capacitive to inductive in this region. From 1 kHz to 3 kHz, the resistance linearly increased to 1.34 Ω while the reactance became more inductive. After 4 kHz, the resistance rose quadratically and achieved a peak value 7 Ω at the previously defined breathing mode frequency of 14.32 kHz whereas the reactance transitioned from smoothly inductive to highly capacitive. An important feature in this analysis is that the net reactance was zero at 14.32 kHz confirming this peak to be a resonant frequency. The reactance declined precipitously from a local maximum of 2.78 Ω at 13.09 kHz to -3.39 Ω at 15.18 kHz demonstrating a strong capacitive effect. Similarly, the resistance indicated a steep drop in this band.

Given the unique resonant structure at approximately 14.32 kHz, we provide an estimate for the capacitance and inductance of the breathing mode. An estimate for the capacitance and inductance of the breathing mode can be obtained by assuming a localized purely capacitive and purely inductive load around the narrow breathing mode band between 10 kHz and 15.8 kHz. First, we assume a purely capacitive reactance when the change in net reactance as a function of frequency, $\frac{\partial X}{\partial f}$, is a minimum. The reactance value associated with, $\text{min}\left(\frac{\partial X}{\partial f}\right)$, is then used to compute the breathing mode capacitance, *C*_*BM*_. The relationship used to estimate the breathing mode capacitance is provided in Eq. 9. For this analysis, the reactance at $\text{min}\left(\frac{\partial X}{\partial f}\right)$ was − 0.875 Ω at 14.49 kHz. Thus, the breathing mode capacitance was estimated to be 12.56 *µ*F.

9 $${C}_{BM}=\frac{1}{2\pi fX\left(\text{min}\left(\frac{\partial X}{\partial f}\right)\right)}$$

A first order estimate for the breathing mode’s inductance, *L*_*BM*_, of the reactive recovery can be attained by assuming a purely inductive rise between the local minimum and local maximum in $\frac{\partial X}{\partial f}$ between 14.4 kHz and 15.8 kHz. The net change in reactance between $X\left({\left.\frac{\partial X}{\partial f}\right|}_{max}\right)$ and $X\left({\left.\frac{\partial X}{\partial f}\right|}_{min}\right)$ divided by $2\pi{f}_{BM}$ yields an estimate for, *L*_*BM*_. For this estimate, *f*_*BM*_ is determined by the zero-crossing of the reactance. The breathing mode inductance was estimated to be 15.3 *µ*H.

For frequencies greater than *f*_*BM*_, the load behaved highly inductively and achieved a second local maximum of 2.11 Ω at 20.55 kHz while resistance continued to reveal a nonlinear behavior. From 20.55 kHz to 28.92 kHz, a mild capacitive drop occurred. The discharge demonstrated prominently inductive characteristics from 29.87 kHz up to 104.33 kHz and attained a maximum value of 6.57 Ω. From 104.33 kHz to 300 kHz, the load was noted to be sharply capacitive. Lastly, the resistance revealed a mostly monotonic increase from 45 kHz to 300 kHz.

### Resistance and reactance of the 6 kW, 20 A operating condition

**Fig. 14:**
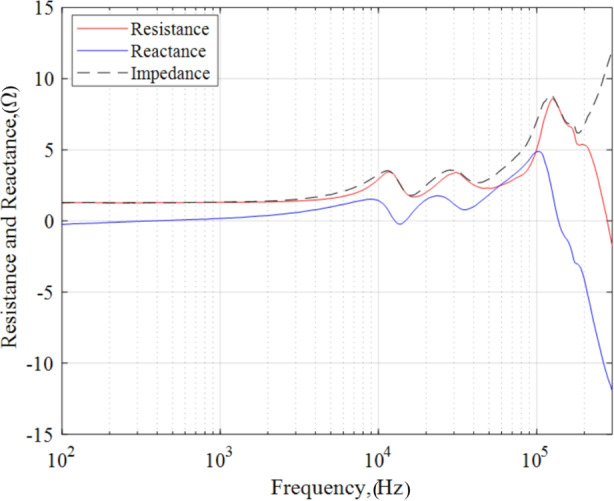
Resistance and reactance at the 6 kW, 20 A test condition

Similarly, we decompose the measured impedance magnitude into its real and imaginary components using the phase information as provided by Eqs. 7 and 8. The real component is associated with resistance whereas the imaginary component is associated with reactance components as described in section “AC analysis and impedance spactroscopy background”. In Fig. 14, we plot resistance in red and reactance in blue for the 6 kW, 20 A discharge operating condition. For reference, we also include the net impedance magnitude in the figure as a dashed curve.

In the band 100 Hz – 1 kHz, the impedance is mostly characterized by a steady resistive value of 1.31 Ω with a small reactive component less than 0.17 Ω. In addition, the reactance was observed to be transitioning from capacitive to inductive in this region. From 1 kHz to 3 kHz, the resistance linearly increased to 1.40 Ω while the reactance became more inductive. After 3.8 kHz, the resistance rose quadratically and achieved a peak value 3.45 Ω at the previously defined breathing mode frequency of 11.43 kHz whereas the reactance transitioned from smoothly inductive to smoothly capacitive. An important feature in this analysis is that the net reactance was approaching zero at 12.67 kHz indicating a potential resonant frequency although the impedance did not achieve a local maximum value. Despite this, we did not identify a discernable resonant structure that would allow us to estimate breathing mode capacitance and inductance values. From 8.95 to 45 kHz, the resistance and reactance undulated with respect to eachother and attained a second peak value of 3.42 Ω at 30.85 kHz and 1.77 Ω at 23.68 kHz, respectively. From 35.09 kHz to 101 kHz, the load behaved inductively and reached a third peak value of 4.88 Ω at 102.99 kHz. The discharge exhibited a strong capacitive behavior from 102.99 kHz to 300 kHz achieving a minimum of -11.76 Ω. Although the reactance also possessed a zero-crossing in this range, the impedance did not achieve a local minimum. The resistance was highly nonlinear and exhibited a third maximum of 8.58 Ω at 125.78 kHz before portraying a steep decline for the remainder of the band.

### Comparison between 4.5 kW, 15 A and 6 kW, 20 A operating conditions

**Fig. 15:**
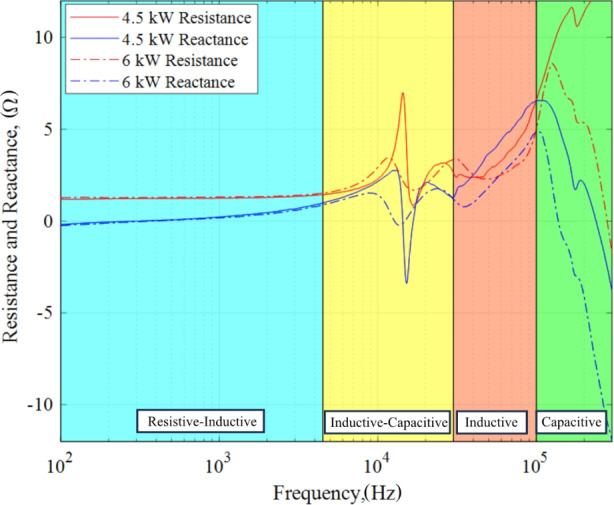
Resistance and reactance at the 4.5 kW, 15 A and 6 kW, 20 A test conditions

The resistive, capacitive, and inductive characteristics of the 4.5-kW and 6-kW discharge operating conditions are contrasted and discussed next. To begin, we plot the resistance and reactance of the 4.5-kW and 6-kW test conditions in Fig. 15. As a visual aid, we provide four shaded regions with labels to identify areas where resistive, capacitive, and/or inductive traits dominated. The two discharge operating conditions generally exhibited similar characteristics across the 100 Hz – 300 kHz band however the resistive, capacitive, and inductive effects were more apparent in the 4.5-kW operating point.

In the 100 Hz – 4.5 kHz band, the 4.5-kW and 6-kW discharge behaved similarly with a relatively constant resistivity. In addition, both profiles displayed a transition to net inductive behavior.

The 4.5 kHz to 30 kHz band was dominated by a capacitive decline and inductive rise around the breathing mode frequency. The 4.5-kW discharge condition exhibited a much stronger capacitance at the breathing mode frequency compared to the smooth dip observed at the 6-kW operating condition. Furthermore, the 4.5-kW impedance profile in the 4.5 –30 kHz band satisfied the conditions akin to parallel or self-induced resonance. Specifically, at *f*_*BM*_ = 14.32 kHz, the measured impedance magnitude was a maximum and the reactance was near zero indicating the voltage and current waveforms were in-phase. Conversely, the 6-kW breathing mode region was milder compared to 4.5-kW and did not readily meet resonance criteria. We can borrow the concept of quality factor, *Q*, to qualitatively explain the possible dynamics in the 4.5 –30 kHz band between the operating conditions. The quality factor is a dimensionless parameter that quantifies the degree of dampness in harmonic oscillators such as parallel RLC circuits. Physically, *Q*, represents the stored energy in reactive power to the real power over one oscillation with frequency, *f*, and is given in Eq. 10. As the energy stored in reactance approaches zero, the parallel RLC circuit exhibits less damping and *Q* increases registering a sharp, narrow peak in impedance. Damping may be achieved by an increase in resistance for a fixed *C*/*L* ratio or by a decrease in inductance for fixed values of *R* and *C*. Based on this, we can argue that if the breathing mode band can be modeled as a parallel RLC circuit, the qualities exhibited by the 6-kW condition may be those of a damped system with decreased inductance as observed in Fig. 15. In either case, the capacitive and inductive characteristics contained in the band 4.5 –30 kHz for both operating conditions cannot be readily described as simple parallel RLC circuits. The main difference between what was measured and the parallel RLC circuit is the highly nonlinear reactance observed at around the *f*_*BM*_ through 30 kHz. Namely, both operating conditions portrayed an inductive rise from the local minimum to a second local maximum that can only be achieved with another combination of RLC elements.

10 $${Q}_{p}=R\sqrt{\frac{C}{L}}$$

The band 30 kHz through 100 kHz is mostly inductive for both test cases. However, the 4.5 kW case exhibited higher inductance of approximately 2 Ω compared to the 6-kW case. The most compelling argument for inductance in this band is the clear, nearly linear rise of reactance countered by a small capacitive effect. In addition, both traces displayed a nonlinear, mostly increasing resistance in this band. The difference in the measured inductance is thought to be related to the self-inductance due to the more energetic Hall current observed in the discharge channel based on the PSD plots for the 4.5-kW case. For instance, *I*_*dis, pk2pk*_ for 4.5 kW was 5.45 A whereas *I*_*dis, pk2pk*_ was measured to be 1.26 A for 6 kW.

Lastly, the 4.5-kW and 6-kW impedance behaved predominantly capacitive from 100 to 300 kHz. However, the rate of decline for the 6-kW case was twice that of the 4.5 kW condition. This difference between the two is further emphasized by the third hump and associated phase transition observed for the 6-kW case as shown previously in Fig. 9. Additionally, the resistive behavior was opposite as the 4.5-kW displayed a mostly increasing trend while the resistance decreased for the 6-kW operating condition.

The main conclusion from this comparative analysis is that the small-signal impedance characteristics are unique to the discharge operating condition even for the same HET design. Indeed, the 4.5 kW discharge operating condition demonstrated more reactive behavior compared to the 6 kW condition. Moreover, these reactive characteristics allow us to describe the HET’s dynamic behavior, such as the breathing mode, in terms of resistance, capacitance, inductance, and resonance.

## Conclusion

Using the concept of impedance spectroscopy and small-signal analysis, we developed an impedance measurement technique to characterize the dynamic behavior of HETs inside a ground-based vacuum test facility. The technique consists of superimposing an excitation voltage signal of known frequency onto the live DC discharge circuit and measuring the current response at that frequency. The excitation voltage and current response measurement were then used to extract the impedance magnitude and phase of the HET discharge about its local DC operating point. This technique was used to measure the impedance profile of a 7-kW HET from 100 Hz to 300 kHz at two thruster operating conditions on krypton: 4.5 kW, 15 A and 6 kW, 20 A. The trends and features present in the impedance magnitude profiles were in agreement with the corresponding PSD plots of *I*_*dis*_(*t*) captured by a high-speed oscilloscope at the two discharge operating conditions. In particular, the breathing mode frequency extracted from the impedance and the PSD profiles were strongly correlated. Thus, the impedance measurement technique may be used as an alternative method to characterize the dynamic characteristics present in HETs.

The impedance magnitude and phase profiles revealed resistive, inductive, and capacitive behavior across different frequency bands. Moreover, these characteristics were unique to each discharge operating condition for the same thruster design. In particular, the 4.5 kW, 15 A test condition showed highly resonant behavior near the breathing mode than the 6 kW, 20 A test condition. As a result, we quantified the capacitance of the breathing mode at the 4.5-kW thruster operating condition to be 12.56 *µ*F with an inductance of 15.3 *µ*H. The 6-kW thruster operating condition did not display the same resonant characteristics at its breathing mode due to its more quiescent-like behavior which was further supported by its relatively low peak-to-peak discharge current value of 1.26 A. Such insights allow us to understand and describe the dynamic characteristics of HETs using electrical engineering concepts.

## Data Availability

Experimental data is available upon request from authors.

## Nomenclature

C: Capacitance, F
*C*_*BM*_: Breathing Mode Capacitance, F
*f*_*BM*_: Breathing Mode Frequency, Hz
*I*_*ac*_: AC current, A
*I*_*dc*_: DC current, A
*I*_*dis*_: Discharge current, A
*I*_*dis, ac*_: AC component of the time-varying discharge current, A
*I*_*dis, dc*_: DC component of the time-varying discharge current, A
*I*_*dis, pk2pk*_: Peak-to-peak value of the time-varying discharge current, A
*I*_*dis, rms*_: Root-mean-square value of the time-varying discharge current, A
*I*_*exc, ac*_: AC current response to the excitation voltage signal of known frequency, A
*L*: Inductance, H
$\dot{m}$: Mass flow rate, mg/s
*R*: Resistance, Ω
*V*_*ac*_: AC voltage source, V
*V*_*dc*_: DC voltage, V
*V*_*dis*_: Discharge voltage, V
*V*_*exc, ac*_: Excitation voltage signal of known frequency and amplitude, V
*V*_*pk*_: Peak amplitude of excitation voltage signal, V
*X*: Reactance, Ω
*X*_*C*_: Capacitive reactance, Ω
*X*_*L*_: Inductive reactance, Ω
*Z*: Impedance, Ω
*Z*_*dis*_: Discharge impedance, Ω
ω: Angular frequency, Hz
*Φ*: Phase shift angle, ˚
